# Elevated Granzyme B in Cytotoxic Lymphocytes is a Signature of Immune Activation in Hemophagocytic Lymphohistiocytosis

**DOI:** 10.3389/fimmu.2013.00072

**Published:** 2013-03-22

**Authors:** Sabine Mellor-Heineke, Joyce Villanueva, Michael B. Jordan, Rebecca Marsh, Kejian Zhang, Jack J. Bleesing, Alexandra H. Filipovich, Kimberly A. Risma

**Affiliations:** ^1^Immunodeficiency and Histiocytosis Program, Division of Bone Marrow Transplantation, Cincinnati Children’s Hospital Medical CenterCincinnati, OH, USA; ^2^Department of Pediatrics, College of Medicine, University of CincinnatiCincinnati, OH, USA; ^3^Division of Human Genetics, Cincinnati Children’s Hospital Medical CenterCincinnati, OH, USA; ^4^Division of Allergy and Immunology, Cincinnati Children’s Hospital Medical CenterCincinnati, OH, USA

**Keywords:** granzyme B, hemophagocytic lymphohistiocytosis, natural killer cells, clinical immunology

## Abstract

Patients with hemophagocytic lymphohistiocytosis (HLH) exhibit immune hyper-activation as a consequence of genetic defects in secretory granule proteins of cytotoxic T lymphocytes (CTL) and natural killer (NK) cells. Murine models of HLH demonstrate significant activation of CTL as central to the disease pathogenesis, but evaluation of CTL and NK activation in children with HLH or inflammatory conditions is not well described. CD8 T cells only express granzyme B (GrB) following stimulation and differentiation into CTL; therefore, we reasoned that GrB expression may serve as a signature of CTL activation. It is unknown whether human NK cells are similarly activated *in vivo*, as marked by increased granule proteins. Perforin and GrB levels are measured in both CTL and NK cells by flow cytometry to diagnose perforin deficiency. We retrospectively compared GrB expression in blood samples from 130 children with clinically suspected and/or genetically defined HLH to age-matched controls. As predicted, CD8 expressing GrB cells were increased in HLH, regardless of genetic etiology. Remarkably, the GrB protein content also increased in NK cells in patients with HLH and decreased following immunosuppressive therapy. This suggests that *in vivo* activation of NK cells occurs in hyper-inflammatory conditions. We conclude that increased detection of GrB in CTL and NK are an immune signature for lymphocyte activation in HLH, irrespective of genetic subtype and may also be a useful measure of immune activation in other related conditions.

## Introduction

Cytotoxic lymphocytes utilize the perforin/granzyme pathway for target-cell killing in both the innate and adaptive immune response (Russell and Ley, 2002; Voskoboinik et al., 2006; Lieberman, 2010). Cytotoxic T lymphocytes (CTL) and natural killer (NK) cells contain specialized secretory lysosomes, cytotoxic granules, which degranulate upon contact with a target-cell. These granules contain perforin (Prf), a membrane-disrupting protein that facilitates the delivery of granzymes, granule proteases that initiate apoptotic death in target-cells.

In humans, genetic defects in the pathway for Prf-mediated lymphocyte cytotoxicity is associated with a primary immunodeficiency presenting with profound systemic inflammation in infancy, familial hemophagocytic lymphohistiocytosis (HLH) (Filipovich, 2009; Risma and Jordan, 2012). Mutations in seven genes have been associated with familial HLH phenotypes (*PRF1*, *UNC13D*, *STX11*, *RAB27A*, *STXBP2*, *SH2D1A*, and *BIRC4*). Five of these genes express proteins (Prf, Munc 13-4, Rab 27a, Syntaxin binding protein 2, and Syntaxin 11) involved in the function of cytotoxic secretory granules, and biallelic genetic mutations lead to impaired Prf-dependent cytotoxicity by impairing Prf function or degranulation of cytotoxic granules (de Saint Basile et al., 2011). The latter two genes (*SH2D1A* and *BIRC4*) encode slam associated protein (SAP) and X-linked inhibitor of apoptosis protein (XIAP) and defects are identified in boys with X-linked lymphoproliferative disease (XLP types 1 and 2), a primary immunodeficiency that may present with HLH, especially upon infection with Epstein Barr Virus (EBV) (Marsh and Filipovich, 2011). SAP deficiency impairs NK and CTL mediated killing of EBV-infected B cells (Cannons et al., 2011). Although clearly associated with HLH (Marsh et al., 2010; Pachlopnik Schmid et al., 2011), the impact of XIAP deficiency on Prf-mediated lymphocyte cytotoxicity remains unclear.

The clinical symptoms of HLH (irrespective of genotype) include fever, lymphadenopathy, jaundice, and mental status changes, and the pathologic signs include pancytopenia, hypercytokinemia, and multisystem organ failure. Hemophagocytosis is frequently seen on bone marrow or liver biopsy, but is variably present on a single biopsy. An immune trigger, such as a viral infection, may or may not be identified. Genetically defined, or primary, HLH is generally fatal unless treated with immune suppression and ultimately bone marrow transplant (Jordan and Filipovich, 2008; Trottestam et al., 2011).

The pathophysiology of HLH has been modeled in *Prf1*-deficient and *Unc13d* deficient mice (Jordan et al., 2004; Crozat et al., 2007). Severe immune dysregulation occurs in response to lymphocytic choriomeningitis virus (LCMV) infection of these genetically defined mice. Jordan and colleagues have shown that the development of HLH phenotype in *Prf1*-deficient mice is dependent upon and defined by dramatic hyper-activation of CTL (Lykens et al., 2011), suggesting that CTL activation may be a reproducible immune signature for HLH. It is unknown whether human CTL and NK are similarly hyper-activated during the clinical course of HLH. Indeed, studies of immune activation in HLH and other systemic inflammatory disorders have focused primarily on non-specific markers of lymphocyte and monocyte activation such as soluble IL-2 receptor alpha (sIL2R or sCD25) and soluble CD163 (Komp et al., 1989; Imashuku et al., 1995; Bleesing et al., 2007; Johnson et al., 2011). Elevated sIL2R levels are utilized as a gold standard, measure of immune activation for HLH, but may be elevated in other lymphoproliferative disorders as well (Bien and Balcerska, 2008). Therefore, we considered whether detection of granule proteins induced in cytotoxic lymphocytes central to the pathogenesis of HLH may herald the immune hyper-activation that is characteristic of HLH.

Granzyme B (GrB) protein levels in human CTL and NK increase following activation *in vitro* (Salcedo et al., 1993; Grossman et al., 2004; Sedelies et al., 2004; Buzza and Bird, 2006; Skak et al., 2008; Kim et al., 2011; Wang et al., 2012). Recent studies have focused on the utility of soluble GrB in the blood as a biomarker of immune activation in humans with a variety of disorders, including autoimmune disease, graft rejection, and viral infection (Spaeny-Dekking et al., 1998; Tak et al., 1999; Goldbach-Mansky et al., 2005; Buzza and Bird, 2006; Altimari et al., 2008; Bem et al., 2008; Truong et al., 2008; Boivin et al., 2009). It is assumed that the elevated blood granzyme levels are due to increased GrB expression by CTL activation; however the contribution from NK cells is unknown. We hypothesized that GrB protein levels in both CD8 T cells (activated CTL) and NK cells would increase secondary to immune activation in patients with HLH. Compared to age-matched controls, we observed that GrB protein expression was increased in both CTL and NK cells of patients with HLH, irrespective of genetic etiology. GrB levels in NK cells also correlated with disease activity. Therefore, human NK cells, like CTL, acquire enhanced GrB protein expression in pro-inflammatory conditions, suggesting a mechanism for up-regulation of cytotoxic function.

## Materials and Methods

### Patients

With approval from the institutional review board (IRB), we retrospectively reviewed clinical data listed on Diagnostic Immunology Laboratory (DIL) test requisition forms, immune testing results, and genetic testing results from patients referred for testing to our center between 2001 and 2011. We defined sub-groups of patients with genetically defined HLH accordingly (see Table 1): (1) FHL2- biallelic *PRF1* mutations, (2) FHL3- biallelic *UNC13D* mutations, (3) FHL5-biallelic STXPB2 mutations, and (4) HLH_xlp_ associated with XLP (mutations in *SH2D1A* or *BIRC4*). Data from five patients with XLP but no evidence of HLH were also compared to boys with HLH_xlp_.

**Table 1:** **Group cohort with HLH**.

Clinical/genetic subtype	Number of patients (*n*) total 130	Age at diagnosis
FHL2 (*PRF1*)	53	1 wk–22 yr
FHL3 (*UNC13D)*	20	3 wk–10 yr
FHL5 (*STXBP2*)	10	12 mo–16 yr
HLH_xlp_ (*SH2D1A* or *BIRC4*)	10	6 wk–17 yr
HLH_unk_	23	2 wk–16 yr
HLH_EBV_	14	3 mo–17 yr

*Wk, week; mo, month; yr, year*.

For comparison, we evaluated a group of patients whose genetic diagnoses were not established HLH_unk_, by reviewing data from over 800 patient blood samples (ages 0–22 years) submitted to this institution’s DIL between 06/2001 and 07/2007 for Prf detection by flow cytometry as part of an evaluation for suspected HLH. Our goal was to utilize stringent criteria for inclusion of patients so as to eliminate samples where the diagnosis of HLH was not confirmed. Therefore, in the HLH_unk_ group we included only patients with a diagnosis of HLH documented by the ordering physician on the sample requisition (or documented following evaluation at our hospital) and an elevated sIL2R. The requirement for elevated sIL2R was not utilized as a criterion for inclusion of patients with documented gene mutations to include the maximum number of children with genetically defined HLH. For the majority of cases, it was not known if a patient had already begun treatment at the time of sample submission. A subset of patients with a clinical history of HLH due to EBV infection documented on the test requisition (HLH_EBV_) and unknown genotype was considered separately (*n* = 14).

In a follow-up analysis, 10 children with HLH were evaluated following and/or during clinical treatment for HLH at this institution. These patients were included if they had repeat testing with both sIL2R and Prf/GrB staining on the same day at a discrete time following treatment. A description of their clinical features is included in Table 2.

**Table 2:** **Granzyme B detection in NK and CTL before and after treatment for HLH, prior to BMT**.

	HLH etiology	Age of onset HLH	SIl2r(pg/ml)	% GrB+ CD8	GrB MCF in NK (783 ± 83)
P1	HLH_EBV_	8yr	137,269	50	2822
			45,231	14	2862
P2	HLH_xlp_ (BIRC4)	6 wk	25,496	96	2509
			9,410	8	1681
P3	HLH_xlp_ (BIRC4)	4 mo	20,748	39	3198
			2,078	4	1231
P4	FHL5	12yr	51,646	36	2312
			883	15	833
P5	FHL_unk_	7 yr	5,323	74	2599
			1,050	30	970
P6	HLH_EBV_	8 yr	12,280	44	2173
			900	4	960
P7	FHL5	2 yr	8,566	49	1670
			1,002	11	1137
P8	FHL2	5 yr	15,647	69	1812
			1,701	98	986
P9	FHL5	4 yr	29,400	81	2048
			656	3	1219
P10	HLH_xlp_ (BIRC4)	16 mo	15888	64	2094
			1876	50	1061

**Figure 1:**
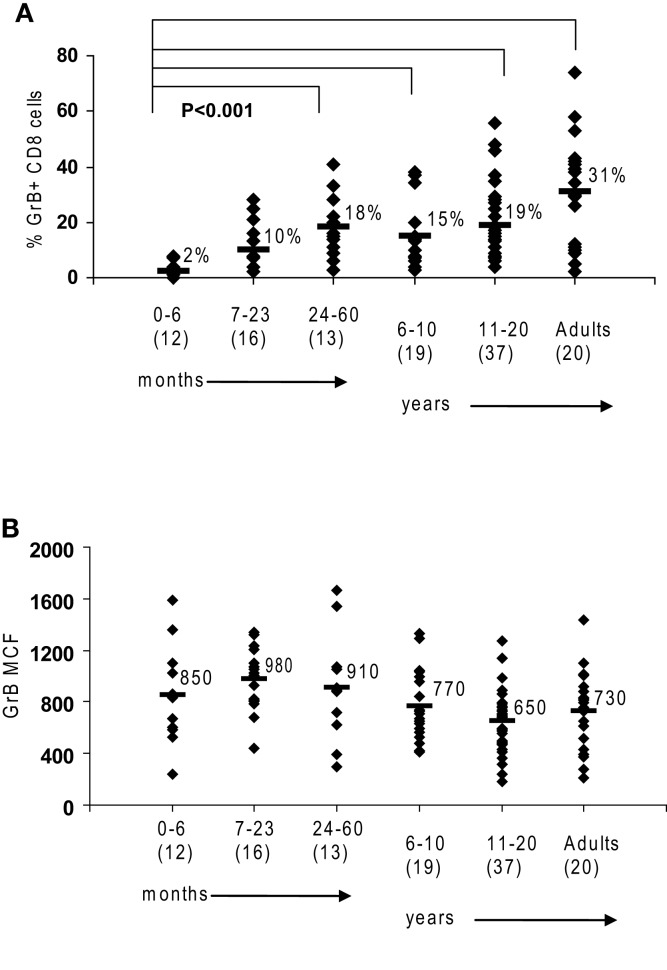
**Granzyme B in human cytotoxic lymphocytes**. Flow cytometry was used to measure protein expression of granzyme B in healthy controls. **(A)** There is an age-dependent increase in CD8 cells expressing granzyme B. **(B)** NK expression of granzyme B as measured by mean channel fluorescence (MCF) does not increase with age. The mean values are listed for each group, with standard deviation. *p* Values were calculated assuming non-parametric analysis. The number of individuals sampled is shown in parentheses under the age range.

*For each patient, serial lab evaluations are displayed by row. Labs that are elevated compared to control values are shaded. The normal control values for sIL2R and GrB in CD8 are age and assay dependent-therefore healthy control values are not listed (See Figure 1 for GrB). The control values for GrB in NK is based on 97 children, aged 0–20 years and represents the 99% confidence interval (parentheses). As >95% of NK cells in patients are GrB (+), only the variable value of MCF is shown*.

### Control group

Samples from healthy children (age 0–20) and adults (>20 years) were obtained and analyzed for GrB expression in NK cells and cytotoxic lymphocytes to establish normal ranges in our laboratory as we previously described for Prf (Kogawa et al., 2002). Residual peripheral blood samples obtained in ethylenediaminetetraacetic acid for complete blood counts (CBC) were retrieved from an outpatient clinic at our institution under an agreement approved by the IRB. The samples included the age and gender of the patient and the result of the CBC. Those samples with CBC or differential parameters outside of the normal range for age were excluded from the study and discarded. All samples were held at room temperature and processed within 24 h of collection.

### Flow cytometric analysis (perforin/granzyme B staining)

**Figure 2:**
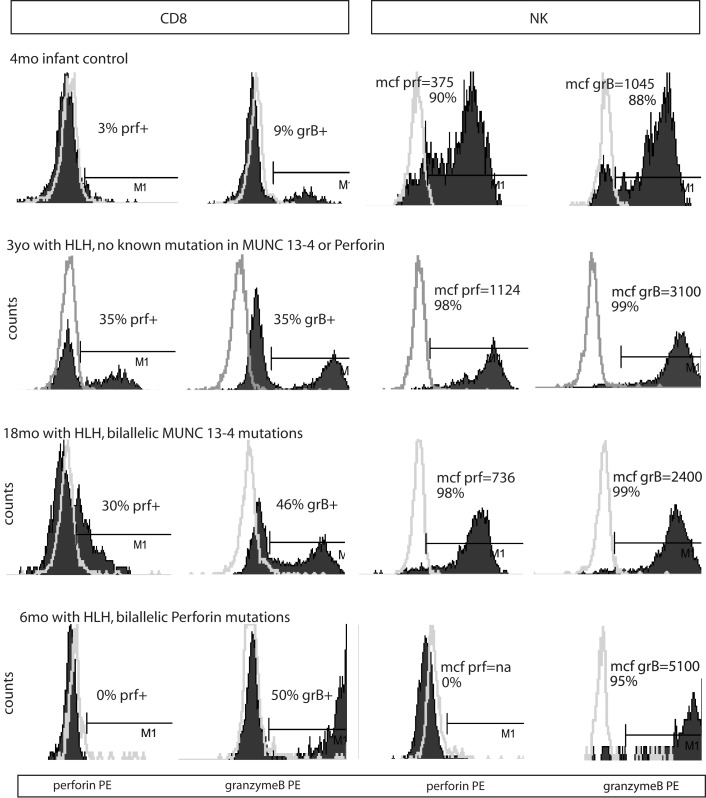
**Representative histograms of flow cytometry for Prf and GrB in patients with HLH**. Dark shading indicates the test antibody. Light gray line is the isotype control.

**Figure 3:**
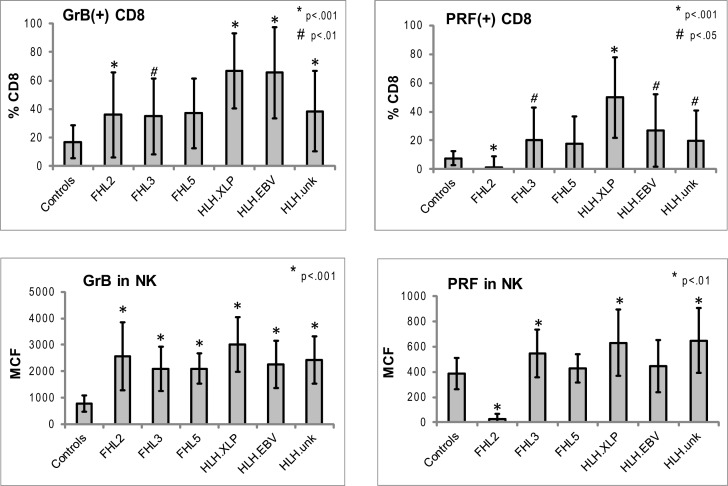
**Expression of Prf and GrB in NK and CD8+ lymphocytes in various subtypes of HLH**. CD8+ cells: shown are the percentages of Prf and GrB positive CD8 cells. GrB and Prf expression was statistically different than controls in all groups except FHL5. For CD8 data, the *p* Values are compared to healthy pediatric controls aged 1–20 years (*n* = 49 for Prf, *n* = 76 for GrB). NK cells: to evaluate the protein content we analyzed the intensity of the fluorescent signal per cell, i.e., MCF. The MCF of GrB was increased in patients with all subtypes of HLH. MCF of Prf was elevated only in patients in HLHxlp, HLH_unk_ and FHL3 groups. For the NK data, pediatric and adult controls were used as there is no age dependence (*n* = 116 for Prf, *n* = 97 for GrB). The error bars represent the standard deviation from the mean. All *p* values assume two tails, non-parametric data.

**Figure 4:**
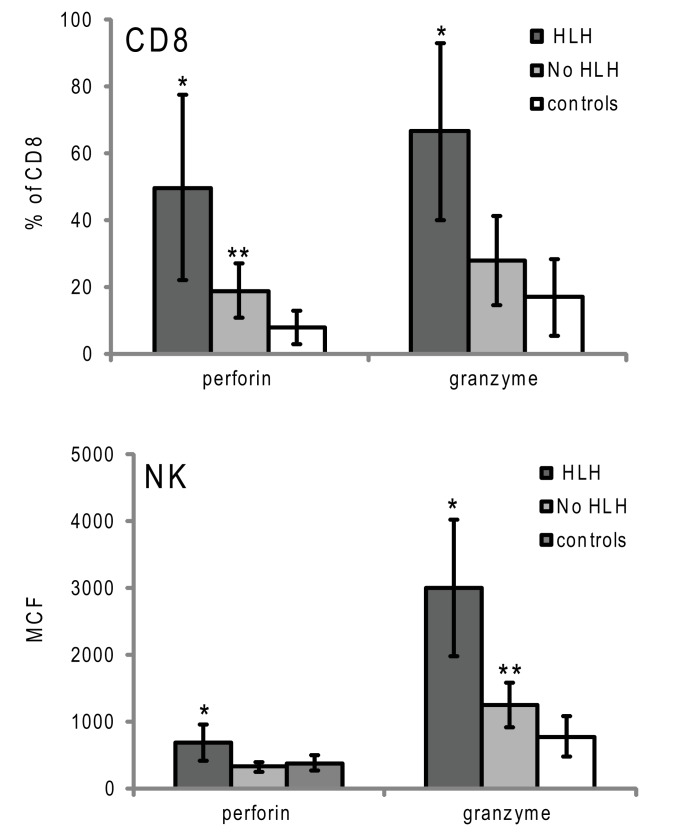
**Granzyme B and perforin expression in cytotoxic lymphocytes from patients with XLP presenting with or without HLH**. Error bars represent standard deviation from the mean. *p < 0.001 compared to no HLH and controls, **p < 0.05 compared to controls.

**Figure 5:**
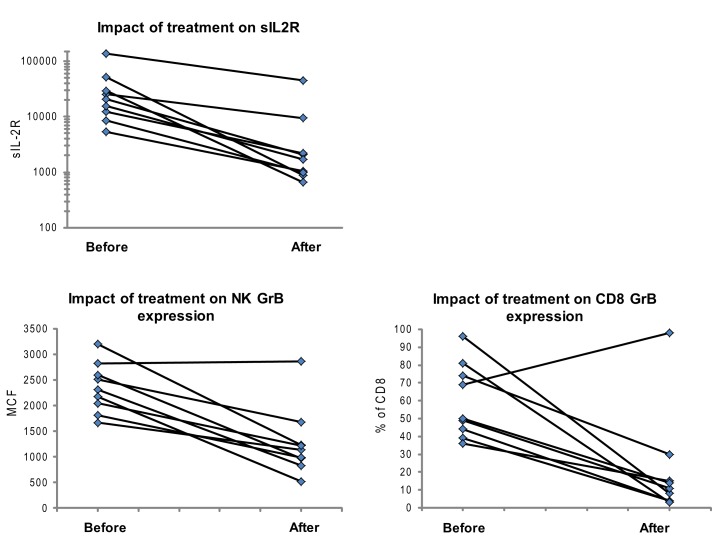
**Granzyme B protein content in cytotoxic lymphocytes is a marker of HLH disease activity**. Ten patients had a follow-up test performed after initiating treatment for HLH (for description of patients see Table 2). Shown are the first and second data points for each patient. Two patients had persistent immune activation, as measured by the gold standard, sIL2R. These two patients also had persistent GrB elevation in the NK compartment (Table 2).

Perforin and GrB staining was performed by using standard procedures as described in previous publications (Kogawa et al., 2002; Molleran Lee et al., 2004). The antibodies used were either PE-conjugated anti-Prf (deltaG9, BD Pharmingen) or PE-conjugated anti-GrB (GB12, Caltag Laboratories, Burlingame, CA, USA). Detection of Prf and GrB expression by flow cytometry in patients and healthy controls was performed using anti-TCRαβ FITC, anti-CD8PerCP, and anti-CD56 APC antibodies (BD) for surface staining and isotype controls for intracellular staining. CD56+ T cells were excluded from the analysis of NK cells. Samples were analyzed using a FACSCalibur or FACSCanto flow cytometer (Becton Dickinson, San Jose, CA, USA). The normal range for the percentage and mean channel fluorescence (MCF) of Prf and GrB in NK cells is age-independent (see Results) and was constant for all daily controls from 2001 to 2009 using FACSCalibur machines. Daily standardization takes place on the flow cytometers using commercial beads with established amounts of fluorescence. With use of the FACSCanto introduced in 2009, a new normal range for the MCF of Prf and GrB was established. Therefore, in the group analysis (Figures 1–4) only samples prior to the change in cytometer were included for NK data. The data from the FACSCanto was included only for the pre/post therapy group (Figure 5). As expected the normal range for the percentages of CD8 cells expressing Prf and GrB did not change from 2001 to 2011.

### Soluble IL 2 receptor

The plasma concentration of the sIL2R was determined using the Human sIL2R (sCD25) ELISA Set (BD OptEIA from BD Biosciences #559104) from 2001 to 2004. From 2004 to 2007, this was changed to a Human sIL2R ELISA Set from R&D. Since February, 2007, the soluble IL2 receptor has been determined using the Immulite 1000 platform, a solid-phase, two-site chemiluminescent immunometric assay system. Soluble sIL2R values were normalized to account for the method utilized. To establish an age-dependent normal range for each method, ethylenediaminetetraacetic acid peripheral blood was obtained from control children of all ages as described above.

### Statistical analysis

Statistical comparisons between two groups were performed using the student *t*-test, assuming two tails and unequal variance as reported in figure legends.

## Results

### Granzyme B expression in cytotoxic lymphocytes from healthy children

We first established the normal ranges for GrB protein expression by flow cytometry using residual blood samples collected from control children of all age groups as previously described. Similar to the observations by Kogawa et al. (2002) that the percentage of CD8 cells expressing Prf increases with age in healthy children, we observed that the percentage of CD 8 cells expressing GrB is very low in young infants and increases with age to an average 31% in adults (*p* < 0.001) (Figure 1A). This is due to the fact that CD8 T cells in a newborn are naive and therefore have not differentiated yet into CTL or memory T cells. With antigen exposure in the first 24 months, there is rapid acquisition of CTL and memory cells, to the same level as adults.

Just as the proportion of Prf-containing NK cells has been reported to be constant from birth to adulthood in humans (Kogawa et al., 2002) we found that GrB expression is detectable in >85% of NK cells at all age groups (not shown). Additionally, the content of GrB in NK cells is constant, with a MCF of 850 in children 0–6-month-old, 730 in adults, and 783 (99% confidence interval is 783 + 83) with all ages combined (obtained in our laboratory between 2001 and 2009) (Figure 1B).

### Granzyme B expression in patients with HLH

We analyzed GrB expression in 93 patients who were identified with known genetic causes of HLH (FHL2, FHL3, FHL5, and HLH_xlp_) (Table 1). In order to determine if patients without clearly established, genetic diagnoses (HLH_unk_) also exhibited the same immune signature, we included 37 additional patients who reported by their physicians as having HLH, of which 14 had EBV-associated HLH (see Table 1). All patients were referred with a clinical diagnosis of HLH by their primary physician and had flow cytometric evaluation of Prf and GrB performed by the DIL.

Typical histograms of Prf and GrB expression in cytotoxic cells are shown in Figure 2 and the results from all patients with HLH summarized in Figure 3. Consistent with our hypothesis, we noted an increased percentage of GrB positive CD8 cells in patients with HLH, in all genetic subtypes. This reached statistical significance for all groups except FHL5, as compared to age-matched healthy normal controls. The lack of statistical significance in FHL5 is due to the lower number of children in this group (*n* = 10), as the mean level was elevated to the same degree as other groups with HLH that did reach a *p* value < 0.01. Prf expression was low (as expected in FHL2 due to biallelic PRF1 mutations), and statistically elevated in all other groups except FHL5. We compared all groups with HLH to controls aged 1–20 years, as the patients with FHL5 were 1 year and older. For the other groups with younger children, we performed an independent analysis including children aged 0–20 years, but this did not change the statistical significance.

We analyzed the MCF of prf and grB in NK cells as a measure of protein content per cell to determine if NK cells similarly upregulated granule proteins. Indeed, GrB content was elevated in NK cells in patients with HLH regardless of genetic subtype. The mean GrB signal (MCF) in NK cells was threefold elevated in all subtypes of HLH, including boys with HLH due to XLP. Therefore, the immunologic phenotype was not dependent upon a granule protein pathway defect *per se*, but instead reflected the common final pathway for immune activation of CTL and NK. Interestingly, the Prf was minimally elevated in some, but not all patients with HLH. It was low, as expected in patients with FHL2 (*PRF1* mutations). As the GrB expression was more consistently (and dramatically) elevated than Prf, we focused on this granule protein as a marker for immune activation.

### Granzyme B expression correlates with immune activation in HLH

Although the data demonstrate that elevated GrB content in NK and an increased percentage of GrB expressing CTLs are present in children with HLH, the correlation of increased GrB detection for immune activation was unclear. To address this, we reviewed data from boys diagnosed with XLP but no evidence of HLH at the time of evaluation. These patients were diagnosed with XLP after a sibling’s diagnosis or after multiple malignancies. As shown in Figure 4, patients with XLP without HLH exhibited subtle increases in GrB detection; whilst the group with HLH had signals that were three times higher than the control group. This data suggests that not only is GrB a signature for immune activation in HLH, but it also increases subtly in individuals with XLP whose baseline immune activation has not yet reached a critical threshold for development of HLH.

We also evaluated whether GrB content decreased with immunosuppression as a second confirmation that protein levels correlated with immune activation. Therefore, we analyzed data from 10 patients who had been treated for HLH at our institution and had at least two samples sent for GrB and Prf testing, along with concomitant sIL2R measurements. All 10 patients had Prf/GrB testing at diagnosis and at a second time point, i.e., during treatment for HLH therapy and prior to bone marrow transplantation. As shown in Table 2, all patients exhibited a decrease in the gold standard for immune activation, sIL2R, during therapy, as expected. The GrB expression in CD8+ cells decreased to normal in 8/10 patients; however, in P8 and P10, GrB expression in CTL remained markedly elevated despite normalization of other markers. Likewise, expression of GrB in NK cells decreased in all but one patient (P1), whose sIL2R remained persistently elevated. Thus, elevations of GrB may serve as a biomarker of immune activation in patients with HLH. Interestingly, although the NK GrB levels dropped, they did not typically normalize, despite a normalized sIL2R. This is reminiscent of the subtle increases in GrB seen in boys with XLP but no evidence of HLH. This suggests that NK expression of GrB may be a more sensitive marker of persistent, dysregulated cytotoxic lymphocyte activation.

## Discussion

Our data in patients with HLH shows for the first time that increased detection of GrB in both CTL and NK are a signature of HLH-associated immune activation, irrespective of genetic etiology. Although expressed constitutively in all human NK cells, GrB protein levels (as measured by flow cytometry) in human NK cells were elevated in patients with HLH and decreased with treatment. This suggests that elevated GrB levels in NK cells may also be a biomarker of disease activity. As a result of this finding, our physicians now consider elevated NK and/or CTL GrB expression as a marker of disease activity, providing additional evidence for evidence of immune activation in the cytotoxic compartment. This is feasible as the diagnostic lab calibrates flow cytometers daily to a fluorescent standard (beads) and defines age-matched ranges prior to testing clinical samples. We also noted increased Prf expressing CD8. In FHL2, due to PRF1 mutations, the Prf levels are generally absent or severely reduced (Figure 3), but in other forms of HLH, PRF levels were also increased in NK cells (FHL3, HLHxlp, and HLHunk).

Occasional samples submitted from patients with HLH exhibited normal levels of GrB expression in cytotoxic lymphocytes. As blood is sent from outside physicians for testing without information regarding treatment, it is likely that these patients had already started immunosuppressive therapy, but this could not be confirmed. Additionally, newborns are occasionally tested prior to the onset of HLH symptomatology if an older sibling has died from HLH. The inclusion of patients with clinically silent disease in our analysis of 130 patients may have led to an underestimation of the maximum GrB expression possible in all subtypes of HLH.

Based on the current understanding of the pathophysiology of HLH, we would predict to find increased Prf/GrB expression in CD8 cells in patients with HLH. The murine model has clearly shown activation of this T cell compartment and CD8 cells acquire both granzymes and Prf during activation and differentiation. As CTL differentiation is dependent upon T cell receptor stimulation, our data implies that antigen presentation via MHC class I is a primary determinant of the immune activation. Viral triggers are presumed in HLH, although not always identified in young infants presenting with HLH. In contrast to CTL activation, elevated NK levels of Prf and GrB have not previously been described under pathologic conditions. Our pre/post evaluation of patients with HLH and the data for individuals with XLP suggests the elevation in these protein markers are a result of immune activation and/or dysregulation.

The finding of NK activation in patients with HLH due to *BIRC4* mutations is interesting in that no clear secretory granule defect has been identified in these patients. It is consistent with the fact that HLH is often a presenting symptom in these patients. (Marsh et al., 2010) Therefore, elevated GrB levels may not simply reflect a failure of secretion or degranulation of this granule associated protein. If this were true, we would expect the highest values to be found in cytotoxic lymphocytes with an inability to degranulate (Munc 13-4 and STXBP2 deficiency). There was no clear difference between sub-groups of HLH. We have not yet addressed the mechanism for increased Prf/GrB detection in NK, but possibilities include increased transcription, post-translational regulation of mRNA stability, increased secretory granule production, and/or increased half-life of the proteins.

Regulation of GrB in NK has been described at the level of mRNA transcription and post-translational regulation through microRNAs, largely through *in vitro* studies of cytokine activation (Kim et al., 2011; Trotta et al., 2011; Wang et al., 2012). As elevated pro-inflammatory cytokines have been described in HLH, it is possible that blood and tissue-derived cytokines drive NK and CTL activation of GrB. It is also likely that ongoing antigenic stimulation (due to a failure to eliminate antigen presenting or virally infected cells) provides an ongoing activating stimulus to NK and CTL. Further studies in murine models of HLH will help clarify these remaining questions.

Although ours is the first study to report GrB protein expression in CTL and NK cells in patients with immune activation due to HLH, GrB transcripts in the blood and GrB ELISPOT measurements of PBMC have been proposed as a biomarker of immune activation to screen for CTL activation leading to graft rejection following solid organ transplants (Altimari et al., 2008; Truong et al., 2008). For example, in patients who received intestinal transplantation, blood monitoring of GrB and Prf RNA were diagnostic markers of acute graft rejection, increasing prior to histological diagnosis of a rejection, and then returning back to baseline with initiation of therapy (Altimari et al., 2008). In addition to investigations following solid organ transplants, studies have evaluated whether GrB levels are elevated in patients with active viral infections such as children with Respiratory Syncytial Virus infection and patients infected with EBV or HIV (Spaeny-Dekking et al., 1998; Bem et al., 2008). Finally, GrB levels in the blood have been associated with disease activity in patients with rheumatoid arthritis (Goldbach-Mansky et al., 2005). No direct comparison have been made of flow cytometry measurements to ELISA or RNA measurements of GrB, making comparisons with these studies challenging to interpret. Evaluation of NK/CTL GrB detection in patients with macrophage activation syndrome (MAS) in patients with juvenile idiopathic arthritis at our center revealed variable results, with increased detection in some, but not all patients with active disease (Grom et al., 2003) and data not shown), highlighting the subtle differences between HLH and MAS.

In summary, we have shown that elevated GrB expression in NK and CTL is a signature of immune activation in patients with HLH regardless of the genetic background. GrB staining of cytotoxic lymphocytes is included routinely as an internal control in the diagnostic immunology lab at our center in the assessment of Prf protein. Our data suggest that it may serve as a useful biomarker of disease activity. As a retrospective study limited to a large cohort of patients with HLH, we have not addressed the specificity of our findings to HLH. Future studies are needed to determine the utility of our findings in other inflammatory conditions such as critically ill patients with systemic inflammatory response syndrome, graft versus host disease, or autoimmune diseases. We would predict that any disorder with profound CTL/NK immune activation secondary to systemic viral activation may share a similar immune profile. Characterization of cytotoxic lymphocyte activation by assessment of GrB detection may thus be a useful tool clinically and in research settings.

## Authors Contribution

Sabine Mellor-Heineke conducted the retrospective chart review, performed data analysis, and wrote the manuscript. Kejian Zhang developed the genetic testing and analyzed the genetic data. Alexandra H. Filipovich, Michael B. Jordan, Jack J. Bleesing, and Rebecca Marsh analyzed the immune data and evaluated the patients treated at this institution. Each of these physicians also contributed to the writing of the manuscript. Joyce Villanueva developed the assays in the DIL and analyzed the immunologic data on patients and controls. Kimberly A. Risma designed the study, interpreted all data, and wrote the manuscript.

## Conflict of Interest Statement

The authors declare that the research was conducted in the absence of any commercial or financial relationships that could be construed as a potential conflict of interest.

## Abbreviations

CMV, cytomegalovirus; CTL, cytotoxic T lymphocytes; EBV, Epstein Barr virus; GrB, granzyme B protein; HLH, hemophagocytic lymphohistiocytosis; MCF, mean channel fluorescence; NK, natural killer; Prf, perforin protein; sIL2R, soluble IL-2 receptor alpha (sCD25).
